# An integrative predictive model for orthokeratology lens decentration based on diverse metrics

**DOI:** 10.3389/fmed.2024.1490525

**Published:** 2024-10-11

**Authors:** Kunhong Xiao, Wenrui Lu, Xuemei Zhang, Shenghua Lin, Jingting Wei, Xiangjie Lin, Qingyuan Cai, Yunxi Ye, Yuan Yao, Jiawen Lin, Li Li

**Affiliations:** ^1^Shengli Clinical Medical College of Fujian Medical University, Fujian Provincial Hospital, Fuzhou University Affiliated Provincial Hospital, Fuzhou, China; ^2^Department of Optometry, School of Medical Technology and Engineering, Fujian Medical University, Fuzhou, China; ^3^School of Basic Medical Sciences, Fujian Medical University, Fuzhou, China; ^4^School of Computer Science and Big Data, Fuzhou University, Fuzhou, China

**Keywords:** orthokeratology, lens decentration, predictive model, Logistic Regression model, myopia

## Abstract

**Purpose:**

To develop a predictive model for orthokeratology (Ortho-K) lens decentration 1 month after wear.

**Methods:**

This study included myopic children who were fitted with Ortho-K lenses at Fujian Provincial Hospital between December 2022 and May 2024. Corneal topography parameters and other relevant metrics were collected pre- and post-treatment. Feature selection was conducted using univariate logistic regression and Lasso regression analysis. A machine learning approach was used to develop multiple predictive models, including Decision Tree, Logistic Regression, Multilayer Perceptron, Random Forest, and Support Vector Machine. Model performance was evaluated using accuracy, sensitivity, specificity, ROC curves, DCA curves, and calibration curves. SHAP values were employed to interpret the models.

**Results:**

The Logistic Regression model demonstrated the best predictive performance, with an AUC of 0.82 (95% CI: 0.69–0.95), accuracy of 77.59%, sensitivity of 85%, and specificity of 61.11%. The most significant predictors identified were age, 8 mm sag height difference, 5 mm Kx1, and 7 mm Kx2. SHAP analysis confirmed the importance of these features, particularly the 8 mm sag height difference.

**Conclusions:**

The Logistic Regression model successfully predicted the risk of Ortho-K lens decentration using key corneal morphological metrics and age. This model provides valuable support for clinicians in optimizing Ortho-K lens fitting strategies, potentially reducing the risk of adverse outcomes and improving the quality of vision for patients. Further validation in clinical settings is recommended.

## 1 Introduction

Myopia has become[126mm] Q9 a significant global public health issue, with the prevalence of myopia and high myopia expected to increase to 4,758 billion and 938 million cases, respectively, by 2050 (1). The number of people with vision impairment due to myopia-related macular degeneration is projected to reach 55.7 million globally, with an estimated 18.5 million expected to be blind (2). Effective control of myopia is crucial for preventing vision impairment. Orthokeratology is a non-surgical method that temporarily reshapes the cornea by wearing rigid gas-permeable contact lenses overnight, allowing for clear vision during the day without the need for glasses (3). It is considered a reliable method for slowing the progression of myopia in children and adolescents (4). However, the potential side effects of Orthokeratology, such as corneal staining, microbial keratitis, and chronic allergic conjunctivitis, have raised concerns among ophthalmologists and parents (5). Lens decentration is a significant risk factor contributing to these complications (6).

The occurrence of lens decentration is associated with multiple factors, among which corneal morphological features play a crucial role. Previous studies have indicated that the degree of lens decentration can be evaluated and quantified using pre-treatment corneal topography parameters (7, 8). Li et al. (9) also noted that eyes with larger corneal elevation differences are often at a higher risk of lens decentration.

Despite these findings, the prediction of lens decentration has predominantly relied on clinical experience and limited parameter analysis, lacking a systematic and quantitative assessment standard. The emergence of machine learning presents an opportunity to create precise predictive models, sometimes even outperforming clinical practitioners (10). Fan et al. (11) developed a machine learning model that improved the accuracy and efficiency of Ortho-k lens fitting, reducing the need for trial lenses and the risk of cross-infection during the pandemic. Similarly, Fang et al. (12) also utilized machine learning models to identify ocular metrics and clinical features for predicting orthokeratology treatment outcomes. Building on these insights, this study aims to develop a model to predict lens decentration 1 month after wear, leveraging corneal morphological features and other relevant metrics. This model will provide a scientific basis for optimizing lens fitting and improving correction strategies in clinical practice.

## 2 Methods

### 2.1 Study population and data collection

This study included myopic children who were fitted with Ortho-k lenses at Fujian Provincial Hospital between December 2022 and May 2024. The inclusion criteria were wearing Ortho-k lenses for at least 1 month and having complete corneal topography and decentration data. Exclusion criteria included corneal abnormalities, a history of ocular diseases before treatment, or inability to cooperate during examinations. All patients were fitted by the same experienced ophthalmologist. None of the participants had undergone orthokeratology treatment prior to this study. This study received approval from the Ethics Committee of Fujian Provincial Hospital (K2024-06-044) and adhered to the principles outlined in the Declaration of Helsinki. All subjects were duly informed and consented to participate in this study.

Before wearing Ortho-k lenses, a series of examinations were conducted, including IOMASTER (Zeiss, Germany), subjective and objective refraction (Phoroptor, Topcon CV-3000, Tokyo, Japan), and slit-lamp evaluation (Suzhou 66 Vision Technology, Suzhou, China). Corneal morphology was assessed using corneal topography (Medmont E300 Topographer, Nunawading, Australia), with each topographic image being automatically captured and optimally focused. The recorded corneal topography parameters included Flat K, Steep K, Kx (Steep K - Flat K), flat eccentricity (E1), steep eccentricity (E2), E ratio (E1/E2), IS Index, Corneal Surface Asymmetry Index, 8 mm sag height difference, Corneal Surface Regularity Index, Central Tear Film Surface Quality, Tear Film Surface Quality, Vertical Q, and Horizontal Q.

**Figure 1:**
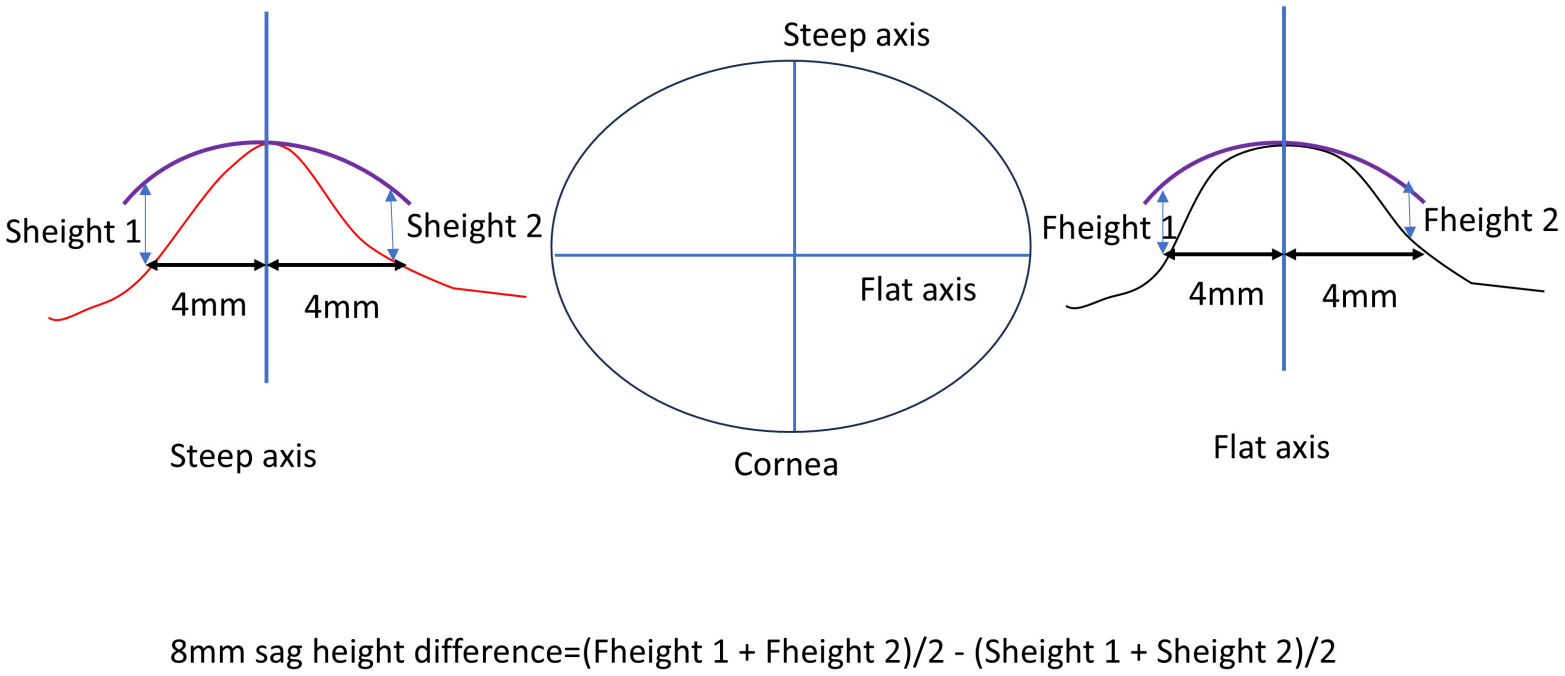
Calculation of the 8 mm sag height difference based on corneal topography.

The 8 mm sag height difference was calculated based on corneal topography maps, specifically using the elevation data. First, the average value in the flat K direction was determined by measuring the corneal height 4 mm from the corneal center on both the nasal and temporal sides. These two values were summed and divided by two to obtain the average flat K direction height. Similarly, the steep K direction height was measured by taking the corneal height 4 mm from the center on both the superior and inferior sides, summing these values, and dividing by two to obtain the average steep K direction height. The difference between these two average values (flat K and steep K directions) represents the 8 mm sag height difference (Figure 1).

Additionally, the curvatures of various corneal regions were measured, including 0–3 mm Corneal Flat Curvature 1, 0–3 mm Corneal Flat Curvature 2, 0–3 mm Corneal Oblique Curvature 1, 0–3 mm Corneal Oblique Curvature 2, 3–5 mm Corneal Flat Curvature 1, 3–5 mm Corneal Flat Curvature 2, 3–5 mm Corneal Oblique Curvature 1, 3–5 mm Corneal Oblique Curvature 2, 5–7 mm Corneal Flat Curvature 1, 5–7 mm Corneal Flat Curvature 2, 5–7 mm Corneal Oblique Curvature 1, and 5–7 mm Corneal Oblique Curvature 2.

The 3 mm Kx1 was defined as the difference between 0 to 3 mm Corneal Flat Curvature 2 and 0 to 3 mm Corneal Flat Curvature 1. The 3 mm Kx2 was defined as the difference between 0 to 3 mm Corneal Oblique Curvature 2 and 0–3 mm Corneal Oblique Curvature 1. The 5 mm Kx1 was defined as the difference between 3 to 5 mm Corneal Flat Curvature 2 and 3 to 5 mm Corneal Flat Curvature 1. The 5 mm Kx2 was defined as the difference between 3 to 5 mm Corneal Oblique Curvature 2 and 3 to 5 mm Corneal Oblique Curvature 1. Finally, the 7 mm Kx1 was defined as the difference between 5 to 7 mm Corneal Flat Curvature 2 and 5 to 7 mm Corneal Flat Curvature 1, while the 7 mm Kx2 was defined as the difference between 5 to 7 mm Corneal Oblique Curvature 2 and 5 to 7 mm Corneal Oblique Curvature 1.

**Figure 2:**
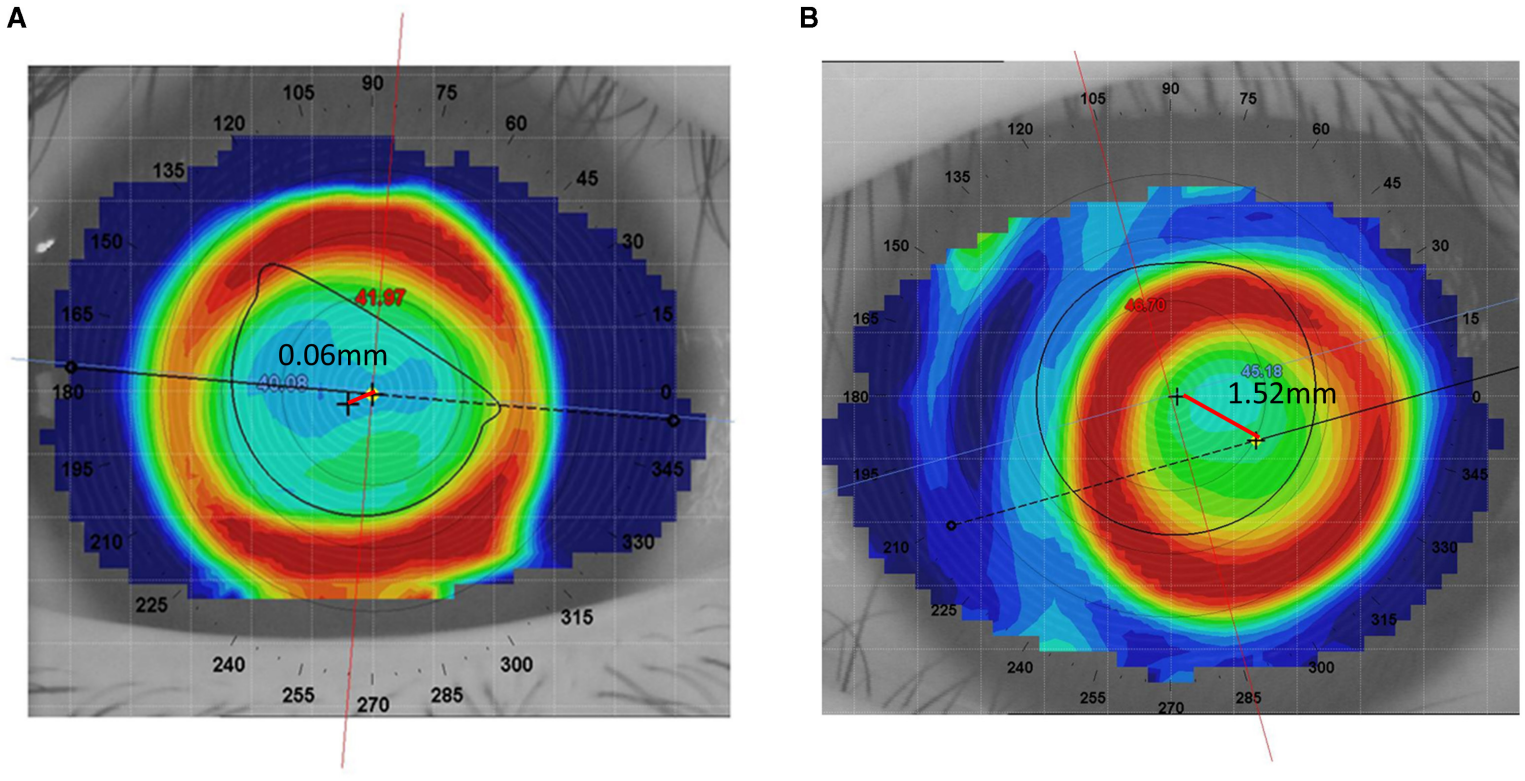
Comparison of well-fitted and decentered orthokeratology lenses. **(A)** Well-fitted Ortho-k lens. **(B)** Decentered Ortho-k lens.

Lens decentration was assessed 1 month after lens wear using a follow-up corneal topography measurement. The difference map was obtained by subtracting the pre-treatment tangential curvature map from the post-treatment tangential curvature map. Following the definition of decentration by Zhang et al. (13), the decentration distance was determined by the topography software, which automatically displayed the distance between the “O point”—the central point of the treatment zone on the cornea, determined by the curvature changes and geometric center created by the orthokeratology lens—and the pupil center. Previous studies suggest that decentration of < 1.0 mm is acceptable, while severe decentration >1.0 mm should be avoided (14, 15). Therefore, in this study, a decentration distance of < 1.0 mm was considered as non-decentration, while a decentration distance >1 mm was considered as decentration. The lens decentration images and well-fitted images are shown in Figure 2.

### 2.2 Lens fitting

The lens fitting procedure in this study followed standard Ortho-K guidelines. We used two brands of Ortho-K lenses based on the CRT design: Lucid (Lucid Korea Co., Ltd., Boston XO material) and ESSENCE (U.S., Paragon HDS^®^ 100 material).

Lens selection was based on corneal curvature, sagittal height, and refractive error, ensuring proper centration and an effective treatment zone during wear. Trial lenses were used to assess the initial fit, and a fluorescein evaluation was performed to check the tear film under the lens. Patients were instructed on proper lens handling, insertion, removal, and care. Follow-up visits were scheduled to monitor lens performance, corneal response, and make any necessary adjustments to the lens fit. All lens parameters for the patients were determined by an experienced attending physician.

### 2.3 Feature selection

To determine the variables included in the machine learning models, univariate analysis was first conducted to assess the differences between patients with and without decentration. Variables with statistical significance (α = 0.05) were then subjected to univariate logistic regression to estimate odds ratios (OR) and 95% confidence intervals (CI). A *p*-value of < 0.05 was considered statistically significant. Additionally, Lasso regression analysis with L1 regularization was used to determine the inclusion and exclusion criteria based on the size of the coefficients. Lasso regression introduces an L1 penalty in the loss function, driving some coefficients to zero, thus enabling automatic feature selection. Variables with coefficients driven to zero in the Lasso regression were excluded, while non-zero variables were retained. The final features included in the model were determined by combining the variables identified through these two models.

### 2.4 Model development and evaluation

Machine learning models were developed using R version 4.4.0 and the Tidymodels package. The data were randomly divided into a training set and a testing set in a 7:3 ratio. Multiple machine learning algorithms were used to build the models (16), including Decision Tree (DT), Logistic Regression, Multilayer Perceptron (MLP), Random Forest (RF), and Support Vector Machine (SVM). Hyperparameter tuning was performed through cross-validation and grid search to achieve optimal model performance. The effectiveness of different algorithms in predicting decentration was compared, and the best-performing model was selected for final analysis. Feature importance analysis was conducted to evaluate the contribution of different features in predicting decentration. Finally, a computational platform was developed using the best-performing model. Model performance was validated on the testing set and evaluated using accuracy, sensitivity, specificity, ROC curves, DCA curves, and calibration curves. For model interpretability, Shapley Additive Planations (SHAP) values were used to explain the model, allowing us to understand the importance and specific impact of each feature in predicting Ortho-k lens decentration.

## 3 Results

### 3.1 Baseline characteristics of the study population

We conducted an initial comparison of baseline characteristics between the lens decentration group (*N* = 66) and the non-decentration group (*N* = 127). In the decentration group, there were 32 males and 34 females, with an average age of (10.55 ± 1.92) years. In the non-decentration group, there were 66 males and 61 females, with an average age of (9.45 ± 1.58) years. A total of 42 variables were included in the study, encompassing demographic information, lens parameters, patient refractive status, and corneal topography parameters (Table 1). The analysis revealed that the decentration group had significantly greater values in age, axial length, spherical equivalent, cylindrical power, steep K, Kx, 3–5 mm corneal oblique curvature 1, 5–7 mm corneal oblique curvature 1, 7 mmkx2, and 8 mm sag height difference compared to the non-decentration group. These findings suggest that these variables may be associated with the risk of Ortho-k lens decentration.

**Table 1:** Comparison of baseline characteristics between patients with and without lens decentration.

**Variable**	**Lens decentration**		***p*-value^b^**
	**No** ***N*** = **127**^a^	**Yes** ***N*** = **66**^a^	
**Age (years)**			<**0.001**
Median (Q1, Q3)	9.00 (8.00, 11.00)	10.00 (9.00, 12.00)	
**Sex (male)**	66 (52%)	32 (48%)	0.6
**Axial length (mm)**			0.012
Median (Q1, Q3)	24.42 (23.92, 25.02)	24.75 (24.27, 25.21)	
**Spherical power (diopters)**			0.002
Median (Q1, Q3)	−1.50 (−2.25, −1.00)	−2.13 (−3.25, −1.50)	
**Cylindrical power (diopters)**			< 0.001
Median (Q1, Q3)	−0.50 (−0.75, −0.25)	−0.75 (−1.25, −0.50)	
**Astigmatism axis**			
Median (Q1, Q3)	165.00 (145.00, 170.00)	170.00 (160.00, 175.00)	0.10
**Jesson factor**			0.3
75	63 (50%)	38 (58%)	
125	64 (50%)	28 (42%)	
**Lens diameter (mm)**			0.3
10	2 (1.6%)	2 (3.0%)	
10.2	22 (17%)	13 (20%)	
10.4	17 (13%)	6 (9.1%)	
10.6	77 (61%)	35 (53%)	
10.8	9 (7.1%)	10 (15%)	
**Flat K (diopters)**			0.9
Median (Q1, Q3)	42.64 (41.85, 43.55)	42.63 (41.79, 43.77)	
**Steep K (diopters)**			0.10
Median (Q1, Q3)	43.59 (42.88, 44.49)	43.85 (43.28, 45.23)	
**Kx (diopters)**			< 0.001
Median (Q1, Q3)	0.97 (0.73, 1.21)	1.33 (1.01, 1.78)	
**Corneal thickness (mm)**			>0.9
Median (Q1, Q3)	530.00 (508.00, 559.00)	534.00 (508.00, 552.00)	
**White to White (mm)**			0.3
Median (Q1, Q3)	12.20 (12.00, 12.50)	12.10 (11.90, 12.40)	
**e1**			0.7
Median (Q1, Q3)	0.65 (0.58, 0.71)	0.66 (0.59, 0.71)	
**e2**			0.4
Median (Q1, Q3)	0.52 (0.42, 0.63)	0.47 (0.34, 0.65)	
**IS Index**			0.4
Median (Q1, Q3)	0.05 (−0.26, 0.38)	−0.04 (−0.35, 0.42)	
**Corneal Surface Asymmetry Index**			0.11
Median (Q1, Q3)	0.58 (0.45, 0.83)	0.63 (0.52, 0.90)	
**Corneal Surface Regularity Index**			0.4
Median (Q1, Q3)	0.51 (0.42, 0.69)	0.50 (0.42, 0.59)	
**Central Tear Film Surface Quality**			0.2
Median (Q1, Q3)	0.05 (0.02, 0.07)	0.04 (0.02, 0.06)	
**Tear film surface quality**			0.11
Median (Q1, Q3)	0.07 (0.05, 0.11)	0.06 (0.04, 0.09)	
**0–3 mm Corneal Flat Curvature 1 (diopters)**			0.9
Median (Q1, Q3)	42.30 (41.70, 43.50)	42.35 (41.60, 43.50)	
**0–3 mm Corneal Flat Curvature 2 (diopters)**			>0.9
Median (Q1, Q3)	42.80 (42.10, 43.70)	42.80 (42.00, 43.90)	
**0–3 mm Corneal Oblique Curvature 1 (diopters)**			0.14
Median (Q1, Q3)	43.90 (43.20, 44.90)	44.20 (43.50, 45.70)	
**0–3 mm Corneal Oblique Curvature 2 (diopters)**			0.3
Median (Q1, Q3)	43.60 (42.80, 44.60)	43.90 (43.00, 44.90)	
**3–5 mm Corneal Flat Curvature 1 (diopters)**			0.9
Median (Q1, Q3)	42.10 (41.40, 43.20)	42.15 (41.20, 43.40)	
**3–5 mm Corneal Flat Curvature 2 (diopters)**			0.6
Median (Q1, Q3)	42.60 (41.80, 43.70)	42.70 (42.10, 43.80)	
**3–5 mm Corneal Oblique Curvature 1 (diopters)**			0.036
Median (Q1, Q3)	43.70 (43.00, 44.50)	44.10 (43.50, 45.50)	
**3–5 mm Corneal Oblique Curvature 2 (diopters)**			0.14
Median (Q1, Q3)	43.20 (42.40, 44.20)	43.50 (42.70, 44.90)	
**5–7 mm Corneal Flat Curvature 1 (diopters)**			0.7
Median (Q1, Q3)	41.40 (40.40, 42.30)	41.45 (40.40, 42.40)	
**5–7 mm Corneal Flat Curvature 2 (diopters)**			>0.9
Median (Q1, Q3)	42.30 (41.70, 43.20)	42.25 (41.80, 43.60)	
**5–7 mm Corneal Oblique Curvature 1 (diopters)**			0.018
Median (Q1, Q3)	43.30 (42.50, 44.10)	43.60 (42.90, 44.90)	
**5–7 mm Corneal Oblique Curvature 2 (diopters)**			0.3
Median (Q1, Q3)	42.80 (42.10, 43.60)	42.95 (42.20, 44.40)	
**Vertical Q**			0.13
Median (Q1, Q3)	−0.53 (−0.63, −0.42)	−0.46 (−0.63, −0.30)	
**Horizontal Q**			0.8
Median (Q1, Q3)	−0.42 (−0.51, −0.35)	−0.42 (−0.50, −0.36)	
**3 mmKx1 (diopters)**			0.5
Median (Q1, Q3)	0.30 (0.10, 0.50)	0.30 (0.10, 0.50)	
**3 mmkx2 (diopters)**			>0.9
Median (Q1, Q3)	0.30 (0.20, 0.50)	0.30 (0.10, 0.50)	
**5 mmkx1 (diopters)**			0.2
Median (Q1, Q3)	0.50 (0.30, 0.80)	0.60 (0.30, 0.90)	
**5 mmkx2 (diopters)**			0.5
Median (Q1, Q3)	0.40 (0.20, 0.60)	0.50 (0.20, 0.80)	
**7 mmkx1 (diopters)**			0.9
Median (Q1, Q3)	0.90 (0.60, 1.50)	1.00 (0.70, 1.30)	
**7 mmkx2 (diopters)**			0.001
Median (Q1, Q3)	0.40 (0.20, 0.70)	0.70 (0.30, 1.00)	
**8 mm sag height difference**			< 0.001
Median (Q1, Q3)	16.00 (10.00, 22.00)	28.00 (21.00, 40.00)	

^a^n (%).

^b^Wilcoxon rank sum test; Pearson's Chi-squared test; Fisher's exact test.

### 3.2 Selection of predictive variables

**Figure 3:**
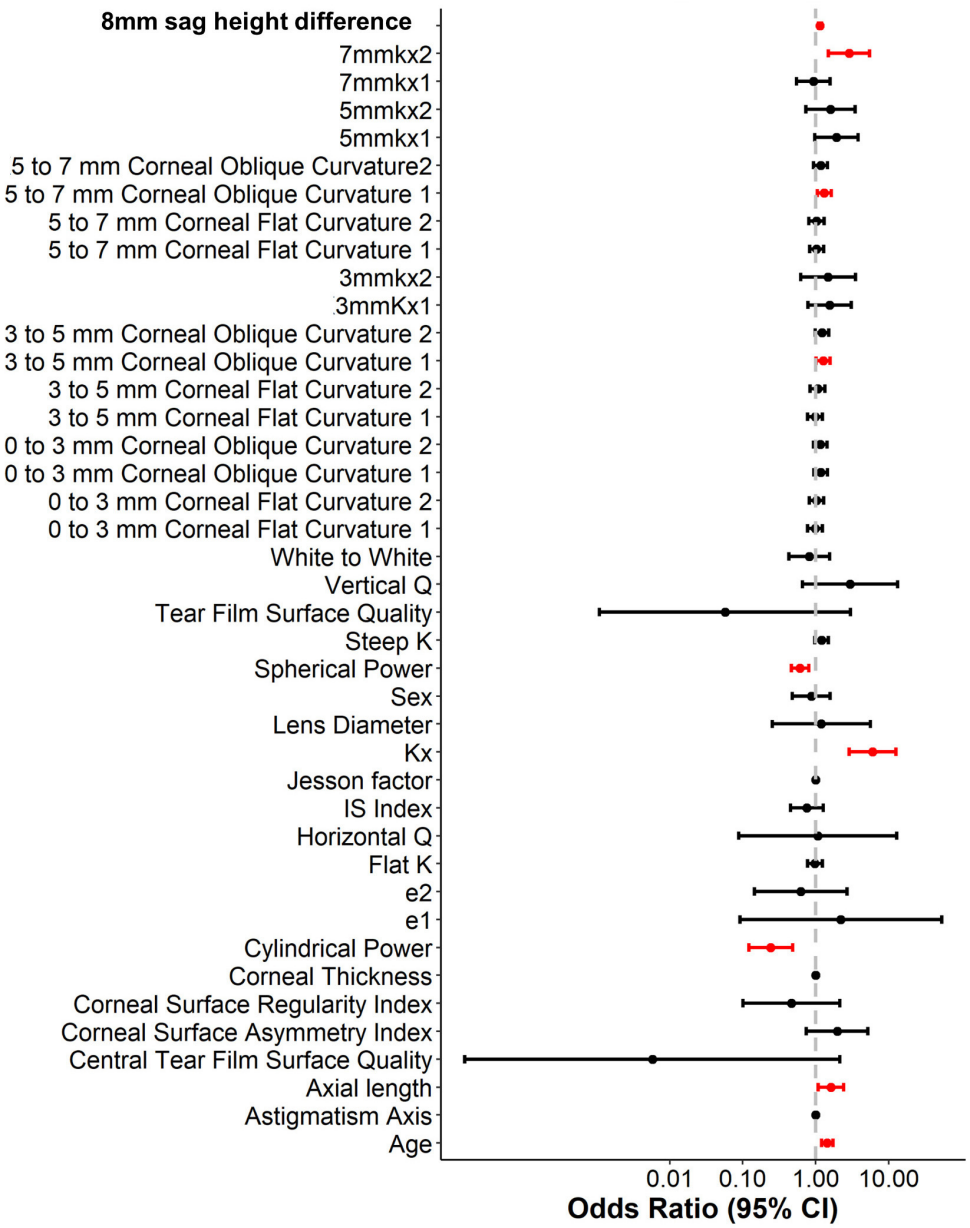
Univariate logistic regression analysis of variables associated with lens decentration.

To enhance the practicality and operability of the model, a univariate logistic regression analysis was conducted on a set of 42 independent variables. The aim was to eliminate redundant variables and identify a more efficient, concise, and accurate set of predictors. The results showed that out of the 20 variables evaluated, only nine were identified as independent predictors of OK lens decentration (Figure 3 and Table 2). Specifically, Spherical Power and Cylindrical Power were identified as independent protective factors, while 3–5 mm Corneal Oblique Curvature 1, Axial Length, 5–7 mm Corneal Oblique Curvature 1, 7 mmkx2, Age, Kx, and 8 mm sag height difference were identified as independent risk factors.

**Table 2:** Univariate logistic regression analysis of variables associated with lens decentration.

**Variable**	**OR**	**Lower CI**	**Upper CI**	***p*-value**
Age	1.440592	1.200426	1.728806	< 0.00001
Sex	0.869875	0.479712	1.577371	0.646169
Axial length	1.622888	1.086496	2.424092	0.018019
Spherical power	0.612215	0.463883	0.807977	0.000528
Cylindrical power	0.242711	0.121778	0.483737	< 0.00001
Astigmatism axis	1.003409	0.998147	1.008699	0.204614
Jesson factor	0.993598	0.981758	1.005581	0.293695
Lens diameter	1.191306	0.253307	5.602714	0.824617
Flat K	0.977877	0.775797	1.232595	0.849769
Steep K	1.206916	0.969956	1.501767	0.091708
Kx	6.02348	2.864692	12.66534	< 0.00001
Corneal thickness	0.998015	0.988983	1.007131	0.668474
White to white	0.815115	0.427562	1.553954	0.534612
e1	2.214293	0.092107	53.23275	0.624135
e2	0.626629	0.144921	2.709493	0.531521
IS index	0.756337	0.450386	1.270121	0.291007
Corneal Surface Asymmetry Index	1.975557	0.746751	5.226409	0.170165
Corneal Surface Regularity Index	0.463113	0.100389	2.136424	0.323727
Central Tear Film Surface Quality	0.005785	1.56E-05	2.148543	0.087882
Tear Film Surface Quality	0.057106	0.001084	3.009506	0.156977
0–3 mm Corneal Flat Curvature 1	0.980719	0.775595	1.240094	0.87082
0–3 mm Corneal Flat Curvature 2	1.02992	0.820136	1.293365	0.79973
0–3 mm Corneal Oblique Curvature 1	1.172542	0.947251	1.451416	0.143687
0–3 mm Corneal Oblique Curvature 2	1.156673	0.9278	1.442004	0.195722
3–5 mm Corneal Flat Curvature 1	0.980365	0.778868	1.233989	0.865852
3–5 mm Corneal Flat Curvature 2	1.062038	0.839585	1.343432	0.615719
3–5 mm Corneal Oblique Curvature 1	1.26399	1.014774	1.574411	0.036538
3–5 mm Corneal Oblique Curvature 2	1.220417	0.980395	1.519201	0.074621
5–7 mm Corneal Flat Curvature 1	1.031232	0.824651	1.289563	0.787434
5–7 mm Corneal Flat Curvature 2	1.021141	0.801836	1.300427	0.865324
5–7 mm Corneal Oblique Curvature 1	1.313607	1.056296	1.633599	0.014191
5–7 mm Corneal Oblique Curvature2	1.16766	0.936689	1.455585	0.168085
Vertical Q	2.949316	0.654758	13.285	0.158982
Horizontal Q	1.065003	0.088312	12.84342	0.96046
3 mmKx1	1.545533	0.780441	3.06067	0.211706
3 mmkx2	1.482172	0.623392	3.524002	0.373182
5 mmkx1	1.93691	0.977514	3.837923	0.058117
5 mmkx2	1.594231	0.734687	3.459396	0.238011
7 mmkx1	0.930556	0.547421	1.581842	0.79033
7 mmkx2	2.863445	1.496853	5.477703	0.001479
8 mm sag height difference	1.148919	1.098197	1.201984	< 0.00001

**Figure 4:**
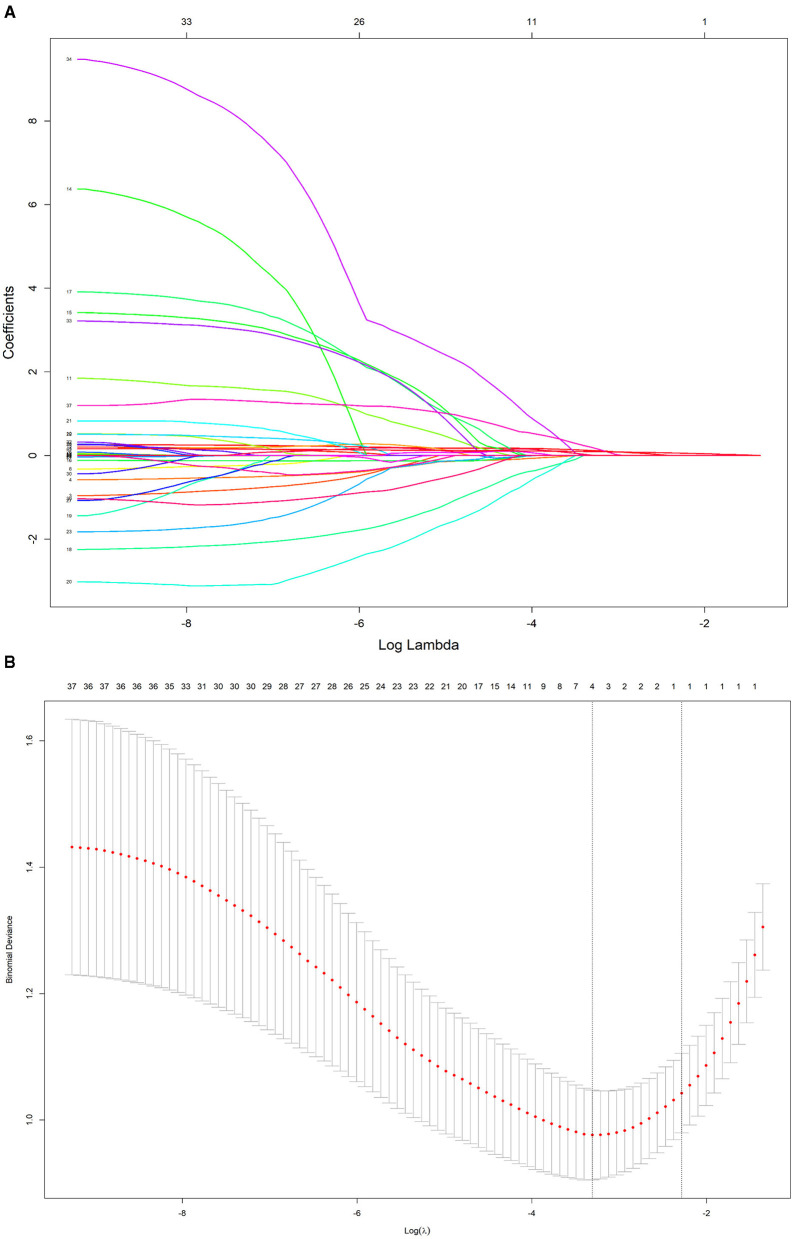
LASSO regression path and cross-validation results. **(A)** LASSO regression path. **(B)** LASSO cross-validation.

To further control for confounding factors, Lasso regression analysis was employed to perform an in-depth selection of the 42 independent variables (Figure 4). Cross-validation revealed that the model achieved optimal fit when four variables were included. The findings indicated that Age, 5 mmkx1, 8 mm sag height difference, and 7 mmkx2 were effective predictive variables. A multicollinearity analysis of these four variables showed that the Variance Inflation Factor (VIF) values were all below 2 (Table 3), indicating that there was no significant multicollinearity among the variables.

**Table 3:** Variance inflation factor (VIF) method checks the multicollinearity of the independent variable.

**Variable**	**VIF**	***p*-value**
Age	1.09	0.006483
5 mmkx1	1.09	0.152453
8 mm sag height difference	1.20	< 0.00001
7 mmkx2	1.21	0.281112

In this study, the intersection of variables identified by logistic regression and Lasso regression includes Age, 5 mm Kx1, 8 mm sag height difference, and 7 mm Kx2. Clinical practice have shown that these parameters effectively reflect the morphological changes in the central and peripheral corneal regions and are crucial for predicting lens decentration. Therefore, we selected them as the final predictive variables for the model.

### 3.3 Comparison of predictive performance across different models

**Figure 5:**
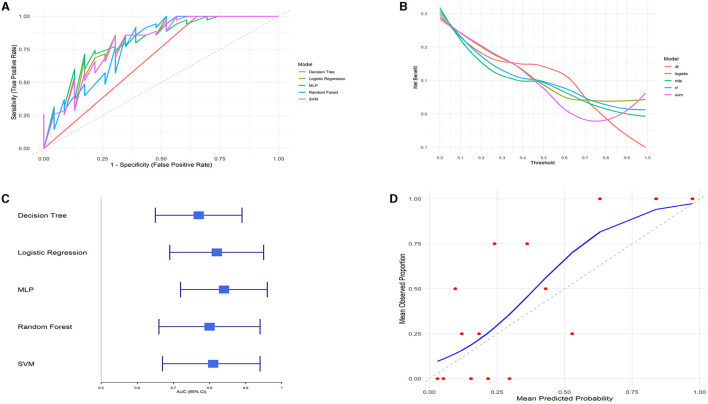
Model performance evaluation. **(A)** ROC curves. **(B)** DCA curves. **(C)** AUC with 95% confidence intervals. **(D)** Calibration curve.

We compared the predictive performance of five different machine learning models, including Decision Tree (DT), Logistic Regression, Multilayer Perceptron (MLP), Random Forest (RF), and Support Vector Machine (SVM). The results of this study indicate that the Logistic Regression model outperformed the other machine learning algorithms, achieving an AUC of 0.82 (95% CI: 0.69–0.95), an accuracy of 77.59%, a sensitivity of 85%, and a specificity of 61.11% (Figures 5A, C and Table 4).

**Table 4:** Different model metrics evaluation.

**Model**	**Accuracy**	**AUC**	**Sensitivity**	**Specifity**
Decision tree	82.75%	0.77 (0.65–0.89)	92.5%	61.11%
Logistic regression	77.59%	0.82 (0.69–0.95)	85%	61.11%
MLP	79.31%	0.84 (0.72–0.96)	85%	66.67%
Random forest	72.41%	0.80 (0.66–0.94)	72.5%	72.2%
SVM	75.86%	0.81 (0.67–0.94)	82.5%	61.11%

The clinical utility of each model at various decision thresholds was evaluated using Decision Curve Analysis (DCA; Figure 5B). The results demonstrate that the Logistic Regression model provided the highest net benefit across most threshold ranges, particularly within the clinically relevant threshold range of 0.2–0.8.

Additionally, we assessed the calibration of the models by comparing the predicted probabilities to the observed event rates using calibration curves. The calibration curve for the Logistic Regression model closely followed the ideal line (where predicted probabilities perfectly match actual outcomes), especially in the mid-probability range, indicating excellent agreement between the predicted probabilities and the observed event rates. Although there was some deviation in the high-probability range, the overall calibration of the Logistic Regression model was good, further confirming the reliability of its predictions (Figure 5D).

Considering all the evaluated metrics, the Logistic Regression model outperformed the other machine learning algorithms in terms of AUC, DCA, and calibration curve performance. This suggests that the Logistic Regression model is highly accurate and robust for this specific task, making it the most suitable predictive tool in this study.

### 3.4 Model interpretability

**Figure 6:**
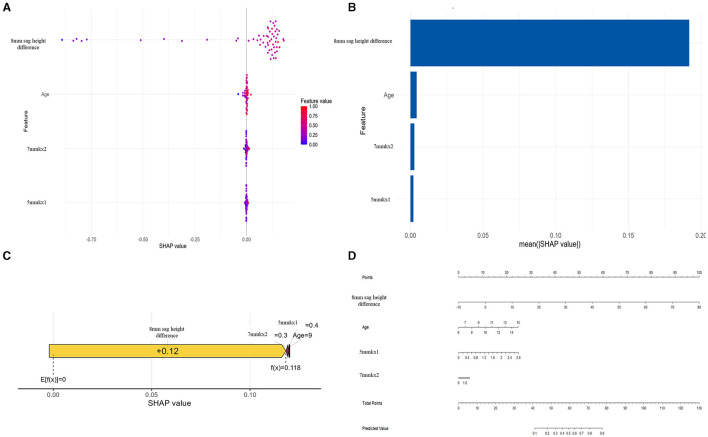
SHAP analysis and nomogram for the Logistic Regression model. **(A)** SHAP Beeswarm plot. **(B)** SHAP feature importance. **(C)** SHAP force plot. **(D)** Nomogram.

To gain deeper insights into the decision-making process of the logistic regression model, we conducted interpretability analysis using SHAP values. The SHAP values of features such as 8 mm sag height difference, 5 mmkx1, 7 mmkx2, and Age are predominantly positive when indicated by red dots, suggesting that higher values of these features tend to increase the predicted probability of lens decentration. Conversely, the blue dots, which represent lower feature values, show negative SHAP values (Figure 6A).

Among all the features, 8 mm sag height difference exhibits the highest mean SHAP value, highlighting its importance as the most influential predictor in the model (Figure 6B). The SHAP force plot illustrates how these features influence the predictive outcomes in a specific individual (Figure 6C). The nomogram further visualizes the contributions of each feature to the predictive outcome in a straightforward manner (Figure 6D). By using the nomogram, it is clear how the scores corresponding to each feature value are summed to predict the overall outcome. The nomogram illustrates how the predicted probability changes with varying values of each feature, emphasizing that 8 mm sag height difference has the greatest impact on the prediction, while other features such as Age, 7 mmkx2, and 5 mmkx1 have relatively smaller effects.

## 4 Discussion

Lens decentration is a significant risk factor for adverse outcomes during ortho-k lens wear. Given that lens decentration can lead to suboptimal vision, corneal damage, and even more severe complications, early identification of high-risk individuals and timely clinical intervention are crucial to reducing adverse events and enhancing the quality of vision for patients (17, 18). Some scholars are also using machine learning technology to make Ortho-K lens fitting more convenient. Koo et al. (19) developed a model for determining comprehensive Ortho-k lens parameters, which can even achieve expert-level accuracy. In this study, we included 42 clinical parameters related to lens decentration and constructed several machine learning models based on these variables. The logistic regression model, incorporating corneal topography parameters (such as 8 mm sag height difference, 5 mmkx1, 7 mmkx2) and age, demonstrated excellent performance in predicting the risk of ortho-k lens decentration. The interpretability of the model was supported by SHAP analysis and the nomogram.

To the best of our knowledge, this is the first study to construct a machine learning model for ortho-k lens decentration using multidimensional corneal morphological parameters (such as 8 mm sag height difference, 5 mm Kx1, and 7 mm Kx2). Previous research has primarily focused on single or limited parameters and lacked the establishment of reliable models. In this study, the Logistic model achieved an AUC of 0.82 (95% CI: 0.69–0.95). Although this is slightly lower than the AUC of 0.84 (95% CI: 0.72–0.96) achieved by the MLP model, the Decision Curve Analysis (DCA) of the Logistic Regression model showed superior performance across multiple threshold ranges, especially in the mid-threshold range (0.3–0.7). The higher net benefit in this range suggests that the Logistic model has greater practical utility in clinical settings. Compared to more complex models, the Logistic Regression model is easier to understand and interpret, which is particularly important for clinical decision-making where model interpretability is critical (20). These suggest that the predictions made by the Logistic Regression model are more practical and can effectively guide clinical decision-making.

The interpretability of predictive models is crucial for their acceptance by clinicians (16). SHAP analysis suggest that higher values of features are associated with a increased probability of decentration. The 8 mm sag height difference had the most substantial impact on the prediction results. It is well-known that the shape of the corneal periphery is crucial for lens fit and stability (21). A larger 8 mm sag height difference indicates significant height variations in the corneal periphery, which could prevent the lens from conforming fully to the corneal surface, increasing the risk of lens displacement during wear. Recent research has also identified the 8 mm sag height difference as an effective predictor of ortho-k lens decentration (14), consistent with our findings. 7 mmkx2 (the oblique curvature difference at 7 mm) and 5 mmkx1 (the flat curvature difference at 5 mm) were also identified as significant predictors in our study. The SHAP values for 7 mmkx2 were clustered around 0, indicating that this feature contributes minimally to the prediction overall. However, in certain cases, higher SHAP values suggest that 7 mmkx2 can significantly influence model output, indicating that patients with significant curvature variation at 7 mm may experience less stable lens fitting. Some data points for 5 mmkx1 had negative SHAP values, indicating that in some cases, 5 mmkx1 might slightly reduce the predicted risk of decentration. Gu et al. (7) suggested that central curvature differences might help support the lens and prevent severe decentration, as eye blinking tends to move the lens up or down during ortho-k lens wear.

Greater age was associated with positive SHAP values, indicating an increased risk of decentration, which has not been previously reported but could be related to changes in sleep patterns. As individuals age, their sleep patterns tend to change significantly. Older individuals often experience lighter sleep and more frequent awakenings (22), which may cause more disturbances to the lens during overnight wear, increasing the likelihood of lens displacement and thus the risk of decentration. Additionally, with age, corneal morphology and biomechanical properties may change, potentially affecting lens fit and stability. Older corneas may be stiffer and less deformable (23), which could contribute to lens decentration. Meanwhile, older children wearing Ortho-K lenses, who are likely transitioning to junior high or high school, may experience reduced lens centration and stability during sleep. This can be attributed to shorter sleep durations and increased academic stress, potentially elevating the risk of lens decentration.

In this study, the nomogram provides a practical application for the predictive model. After obtaining the key parameters, clinicians can find the corresponding score for each parameter on the “Points” scale. By summing the scores for each parameter, a total score is obtained. Based on the total score, clinicians can determine the predicted risk on the “Predicted Value” scale. If a patient has a higher predicted risk, the clinician can take appropriate interventions based on the model's results, such as adjusting the lens design parameters (e.g., increasing the back optic zone or diameter), increasing the frequency of follow-ups to monitor lens positioning, or implementing other measures to reduce the risk of decentration.

Despite the meaningful findings of this study, there are some limitations. First, the sample size was relatively small, which might affect the generalizability of the model. Future studies should include a larger sample size and consider incorporating additional clinical variables to further improve the predictive accuracy of the model. Second, this study primarily focused on the development and preliminary validation of the model, and it has not yet been widely applied and validated in real-world clinical settings. Subsequent research should evaluate its applicability and effectiveness in different populations. Finally, the imbalance in sample size between the decentration and non-decentration groups, may lead to better model fitting for the non-decentration group during training, potentially affecting the accuracy of predictions for patients in the decentration group. Future research should aim to increase the sample size of the decentration group or apply weighting methods to correct for this imbalance, enhancing the model's predictive performance and its applicability in clinical practice.

## 5 Conclusion

This study successfully developed a Logistic-based predictive model to assess the risk of lens decentration in ortho-k lens wear. The findings indicate that corneal morphological metrics (8 mm sag height difference, 5 mmkx1, 7 mmkx2) and age are key predictive factors influencing lens decentration. This model provides valuable data support for clinicians during the ortho-k lens fitting process, aiding in the prediction of decentration risk. Consequently, it can help optimize personalized fitting strategies and reduce the occurrence of lens decentration.

## Data Availability

The original contributions presented in the study are included in the article/supplementary material, further inquiries can be directed to the corresponding author.
